# Supplementary material for: DENR promotes translation reinitiation via ribosome recycling to drive expression of oncogenes including ATF4
Source: Nat Commun. 2020 Sep 16;11:4676. doi: 10.1038/s41467-020-18452-2 (PMC7494916; doi:10.1038/s41467-020-18452-2)
Supplement: Supplementary file 1 — Supplementary Information [file 41467_2020_18452_MOESM1_ESM.pdf]

Supplementary Information

DENR promotes translation reinitiation via ribosome recycling

to drive expression of oncogenes including *ATF4*

Bohlen et al.

CONTENTS

1) Supplementary Tables..p. 2

2) Supplementary Figures and Legends..p. 6

1) SUPPLEMENTARY TABLES

Supplementary Table 1: Cloning Oligos

Name	Sequence	Purpose
OJB0015	caccgCTTCGAGTCCTTTATTGTGG	sgRNA DENR 1 (Used to generate DENRKO1)
OJB0016	aaacCCACAATAAAGGACTCGAAGc	sgRNA DENR 2 (Used to generate DENRKO2)
OJB0017	caccgAAGGACTCGAAGTGGGTAAT	sgRNA MCTS1 1
OJB0018	aaacATTACCCACTTCGAGTCCTTc	sgRNA MCTS1 2
OJB0058	ccggaagctt tttctactttgcccgcccacagATG	ATF4 5'UTR Forward
OJB0059	ccggATCGATgtCATggtgga ttcagcccccaaacccgactggtcg	ATF4 5'UTR Reverse
OJB0056	ccggaagcttACCGCGCACAAGGCCCTGCGGTGGG	Drosha 5'UTR Forward
OJB0057	ccggTTCGAAgtCATggtggTCCGCCTGGATATGTCACATCTTCC	Drosha 5'UTR Reverse
OJB0062	ccggaagctt agttccaagtttggagcttttagc	MAP2K6 5'UTR Forward
OJB0063	ccggTTCGAAgtCATggtgg cctttcctttgatgggggaggggag	MAP2K6 5'UTR Reverse
OJB0070	ccggaagctttttctactttgcccgcccacagATGTCCTCCTCCTAGttttctctgcgc	ATF4 Extend uORF1: Forward
OJB0072	ccggaagctt tttctactttgcccgcccacagTAC TAGttttctctgcgc	ATF4 Delete uORF1: Forward
OJB0074	tccacggccaccATG TCCTCCTCC GCGTATTAGgggcag	ATF4 Extend uORF2 Forward
OJB0075	ctgcccCTAATACGCGGAGGAGGACATggtggccgtgga	ATF4 Extend uORF2 reverse
OJB0076	tccacggccaccTAC GCGTATTAGgggcag	ATF4 Delete uORF2 Forward
OJB0077	ctgcccCTAATACGC GTAggtggccgtgga	ATF4 Delete uORF2 reverse
OJB0078	ccagcggcttaagcc TAC gcgcttctcacggca	ATF4 Delete uORF3 Forward
OJB0079	tgccgtgagaagcgcGTAggcttaagccgctgg	ATF4 Delete uORF3 reverse
OJB0145	ccggaagctt agaatcggagagccggtggc	HinD III c-Raf 5'UTR Forward
OJB0146	ccggTTCGAAgtCATggtgg cagcttaaacaattcttaaa	c-Raf 5'UTR - BSP119l Reverse
OJB0147	ccggaagctt aagagaggcccaagatggag	HinD III a-Raf 5'UTR Forward
OJB0148	ccggTTCGAAgtCATggtgg cttagattttgtcaagatgg	a-Raf 5'UTR - BSP119l Reverse
OJB0149	ccggaagctt gtcgccagcagctggagcgga	HinD III PIK3R1 5'UTR Forward
OJB0150	ccggTTCGAAgtCATggtgg caaatctgctaccatgcaac	PIK3R1 5'UTR - BSP119l Reverse
OJB0151	ccggaagctt aggccgctcggaaccgtggc	HinD III PIK3R2 5'UTR Forward
OJB0152	ccggTTCGAAgtCATggtgg cgtgagtggggtggggtctg	PIK3R2 5'UTR - BSP119l Reverse
OJB0153	ccggaagctt aagagaggcccaagTAGgagacggcggcggctgtagc	a-RAF uORF1 mut Forward
OJB0154	cgtgacaggagccccTAGgcacctgcccagccc	a-RAF uORF2 mut Forward
OJB0155	gggctgggcaggtgcCTAggggctcctgtcacg	a-RAF uORF2 mut Reverse
OJB0156	gggccgccatcttagTAGgcgggagtaagagga	c-RAF uORF1 mut Forward
OJB0157	tcctcttactcccgcCTActaagatggcggccc	c-RAF uORF1 mut Reverse
OJB0158	agcgcgtttgctacgTAGcgggggctgctcggg	c-RAF uORF2 mut Forward
OJB0159	cccgagcagcccccgCTAcgtagcaaacgcgct	c-RAF uORF2 mut Reverse
OJB0160	ctggggacgcgccgaTAGtgaccgcctcccgct	c-RAF uORF3 mut Forward
OJB0161	agcgggaggcggtcaCTAtcggcgcgtccccag	c-RAF uORF3 mut Reverse
OJB0176.1	CTAGTgtccacggccaccATGGCGTATTAGgggcagA	ATF4 uORF2 in synthetic context forward
OJB0177	CCGGTctgcccCTAATACGCCATggtggccgtggacA	ATF4 uORF2 in synthetic context reverse
OJB0178.1	CTAGT GTGtccggccaccATGGCGTATTAGTCCTCC A	ATF4 uORF2 in synthetic context forward no context
OJB0179	CCGGT GGAGGACTAATACGCCATggtggccggaCAC A	ATF4 uORF2 in synthetic context reverse no context
OJB0180.1	CTAGT gtccacggacaaaATGGCGTATTAGgggcag A	ATF4 uORF2 in synthetic context forward no Kozak
OJB0181	CCGGT ctgcccCTAATACGCCATtttgtccgtggac A	ATF4 uORF2 in synthetic context reverse no Kozak
OJB0182.1	CTAGT gtccacggccaccATGTCCTCCTAAgggcag A	ATF4 uORF2 in synthetic context forward AA seq mut
OJB0183	CCGGT ctgcccTTAGGAGGACATggtggccgtggac A	ATF4 uORF2 in synthetic context reverse AA seq mut
OJB0184.1	CTAGT gtccacggccaccATGGCGTATTAAgggcag A	ATF4 uORF2 in synthetic context forward no stop codon
OJB0185	CCGGT ctgcccTTAATACGCCATggtggccgtggac A	ATF4 uORF2 in synthetic context reverse no stop codon
OJB0186	CTAGTaagagaggcccaagATGgagacggcggcggctgtagcggcgtgacaggagccccA	aRAF uORF1 in synthetic context forward
OJB0187	CCGGTggggctcctgtcacgccgctacagccgccgccgtctcCATcttgggcctctcttA	aRAF uORF1 in synthetic context reverse
OJB0238	CTAGT aagagaggcccaagATGTCCTCCTCCTCCgctgtagcggcgtgacaggagccccA	aRaf uORF2 first half AA-seq mutated F
OJB0239	CCGGTggggctcctgtcacgccgctacagcGGAGGAGGAGGACATcttgggcctctcttA	aRaf uORF2 first half AA-seq mutated R

OJB0240	CTAGT aagagaggcccaagATGgagacggcggcgTCCTCCTCCTCCTCCTAAcaggagccccA	aRaf uORF2 second half AA-seq mutated F
OJB0241	CCGGTggggctcctgTTAGGAGGAGGAGGAGGAcgccgccgtctcCATcttgggcctctcttA	aRaf uORF2 second half AA-seq mutated R
OJB0242	CTAGTaagagaggcccaagATGTCCTCCTCCTCCTCCTCCTCCTCCTCCTAAcaggagccccA	aRaf uORF2 AA-seq mutated F
OJB0243	CCGGTggggctcctgTTAGGAGGAGGAGGAGGAGGAGGAGGAGGACATcttgggcctctcttA	aRaf uORF2 AA-seq mutated R
OJB0244	ctagtGTGtccggacaaaATGTCCTAATCCTCCa	2 AA uORF Serine F
OJB0245	ccggtGGAGGATTAGGACATtttgtccggaCACa	2 AA uORF Serine R
OJB0246	Ctagt GTGtccggacaaaATGGCGTAATCCTCC a	2 AA uORF Alanine F
OJB0247	Ccggt GGAGGATTACGCCATtttgtccggaCAC a	2 AA uORF Alanine R
OJB0248	Ctagt GTGtccggacaaaATGTATTAATCCTCC a	2 AA uORF Tyrosine F
OJB0249	Ccggt GGAGGATTAATACATtttgtccggaCAC a	2 AA uORF Tyrosine R
OJB0327	CTAGT aagagaggcacaaaATGgagacggcggcggctgtagcggcgtgacaggagcccc A	aRAF U1 mutate Kozak F
OJB0328	CCGGT ggggctcctgtcacgccgctacagccgccgccgtctcCATtttgtgcctctctt A	aRAF U1 mutate Kozak R
OJB0329	CTAGT aagagaggcccaagATGgagacggcggcggctgtagcggcgtgaTCCTCCTCCTC A	aRAF U1 mutate Stop context F
OJB0330	CCGGT GAGGAGGAGGAtcacgccgctacagccgccgccgtctcCATcttgggcctctctt A	aRAF U1 mutate Stop context R
OJB0331	CTAGT aagagaggcccaagATGgagacggcggcggctgtagcggcgtaacaggagcccc A	aRAF U1 mutate Stop F
OJB0332	CCGGT ggggctcctgttacgccgctacagccgccgccgtctcCATcttgggcctctctt A	aRAF U1 mutate Stop R
OJB0333	CTAGT aagagaggcccaagATGgagacggcggcgTCCgtagcggcgtgacaggagcccc A	aRAF U1 mutate AA-4 F
OJB0334	CCGGT ggggctcctgtcacgccgctacGGAcgccgccgtctcCATcttgggcctctctt A	aRAF U1 mutate AA-4 R
OJB0335	CTAGT aagagaggcccaagATGgagacggcggcggctTCCgcggcgtgacaggagcccc A	aRAF U1 mutate AA-3 F
OJB0336	CCGGT ggggctcctgtcacgccgcGGAagccgccgccgtctcCATcttgggcctctctt A	aRAF U1 mutate AA-3 R
OJB0337	CTAGT aagagaggcccaagATGgagacggcggcggctgtaTCCgcgtgacaggagcccc A	aRAF U1 mutate AA-2 F
OJB0338	CCGGT ggggctcctgtcacgcGGAtacagccgccgccgtctcCATcttgggcctctctt A	aRAF U1 mutate AA-2 R
OJB0339	CTAGT aagagaggcccaagATGgagacggcggcggctgtagcgTCCtgacaggagcccc A	aRAF U1 mutate AA-1 F
OJB0340	CCGGT ggggctcctgtcaGGAcgctacagccgccgccgtctcCATcttgggcctctctt A	aRAF U1 mutate AA-1 R
OJB0383	ccggaagctt acgacgggggaggtgctgta	TSC1 5'UTR Forward
OJB0384	ccggTTCGAAgtCATggtgg cgctcgaaggcgctgtgctg	TSC1 5'UTR Reverse
OJB0387	ccggaagctt aggcggggcagccccggtag	CUL1 5'UTR Forward
OJB0388	ccggTTCGAAgtCATggtgg gggatgttctaaagtcgtcc	CUL1 5'UTR Reverse
OJB0389	ccggaagctt agcttgcggcctgtgtctat	CDK4 5'UTR Forward
OJB0390	ccggTTCGAAgtCATggtgg agatcaagggagaccctcac	CDK4 5'UTR Reverse
OJB0395	Ctagt GTGtccggacaaaATG GCG GCG GCGTAATCCTCC a	Forward 4AA GCG uORF
OJB0396	Ccggt GGAGGATTACGCCGCCGCCATtttgtccggaCAC a	Reverse 4AA GCG uORF
OJB0397	Ctagt GTGtccggacaaaATG GGC GGC GGC TAATCCTCC a	Forward 4AA GGC uORF
OJB0398	Ccggt GGAGGATTAGCCGCCGCCCATtttgtccggaCAC a	Reverse 4AA GGC uORF
OJB0399	Ctagt GTGtccggacaaaATG CGG CGG CGG TAATCCTCC a	Forward 4AA CGG uORF
OJB0400	Ccggt GGAGGATTACCGCCGCCGCATtttgtccggaCAC a	Reverse 4AA CGG uORF
OJB0404	Ctagt GTGtccggacaaaATG CTG CTG CTG TAATCCTCC a	Forward 4AA CTG uORF
OJB0405	Ccggt GGAGGATTACAGCAGCAGCATtttgtccggaCAC a	Reverse 4AA CTG uORF
OJB0406	Ctagt GTGtccggacaaaATG TCC TCC TCC TAATCCTCC a	Forward 4AA S-TCC uORF
OJB0407	Ccggt GGAGGATTAGGAGGAGGACATtttgtccggaCAC a	Reverse 4AA S-TCC uORF
OJB0408	Ctagt GTGtccggacaaaATG GCC GCC GCC TAATCCTCC a	Forward 4AA A-GCC uORF
OJB0409	Ccggt GGAGGATTAGGCGGCGGCCATtttgtccggaCAC a	Reverse 4AA A-GCC uORF
OJB0410	Ctagt GTGtccggacaaaATG GCT GCT GCT TAATCCTCC a	Forward 4AA A-GCT uORF
OJB0411	Ccggt GGAGGATTAAGCAGCAGCCATtttgtccggaCAC a	Reverse 4AA A-GCT uORF
OJB0412	Ctagt GTGtccggacaaaATG GCA GCA GCA TAATCCTCC a	Forward 4AA A-GCA uORF
OJB0413	Ccggt GGAGGATTATGCTGCTGCCATtttgtccggaCAC a	Reverse 4AA A-GCA uORF
OJB0414	Ctagt GTGtccggacaaaATG GGT GGT GGT TAATCCTCC a	Forward 4AA G-GGT uORF
OJB0415	Ccggt GGAGGATTAACCACCACCCATtttgtccggaCAC a	Reverse 4AA G-GGT uORF
OJB0416	Ctagt GTGtccggacaaaATG CCG CCG CCG TAATCCTCC a	Forward 4AA P-CCG uORF
OJB0417	Ccggt GGAGGATTACGGCGGCGGCATtttgtccggaCAC a	Reverse 4AA P-CCG uORF
OJB0418	Ctagt GTGtccggacaaaATG CAT CAT CAT TAATCCTCC a	Forward 4AA H-CAT uORF
OJB0419	Ccggt GGAGGATTAATGATGATGCATtttgtccggaCAC a	Reverse 4AA H-CAT uORF
OJB0420	Ctagt GTGtccggacaaaATG GAA GAA GAA TAATCCTCC a	Forward 4AA E-GAA uORF
OJB0421	Ccggt GGAGGATTATTCTTCTTCCATtttgtccggaCAC a	Reverse 4AA E-GAA uORF
OLH035	ccggATCGATgtCATggtggaGGTGGCCGTGGACCCTGAGG	Cloning of ATF4 5´UTR only uORF1 (+sequence until uORF2)_reverse
OLH036	ccggATCGATgtCATggtggaGGCTTAAGCCGCTGGGGGTT	Cloning of ATF4 5´UTR uORF1 and uORF2 (+sequence until uORF3)_reverse
OLH45	ctagtGTGtccggacaaaATGTAATCCTCCa	Cloning stuORF reporter with 1aa F
OLH46	ccggtGGAGGATTACATtttgtccggaCACa	Cloning stuORF reporter with 1aa R
OLH47	ctagtGTGtccggacaaaATGTCCTCCTAATCCTCCa	Cloning stuORF reporter with 3aa F
OLH48	ccggtGGAGGATTAGGAGGACATtttgtccggaCACa	Cloning stuORF reporter with 3aa R

OLH49	ctagtGTGtccggacaaaATGTCCTCCTCCTCCTCCTCCTCCTAATCCTCCa	Cloning stuORF reporter with 8aa F
OLH50	ccggtGGAGGATTAGGAGGAGGAGGAGGAGGAGGACATtttgtccggaCACa	Cloning stuORF reporter with 8aa R
OLH51	ctagtGTGtccggacaaaATGTCCTCCTCCTCCTCCTCCTCCTCCTCCTCCT CCTCCTCCTCCTCCTCCTCCTCCTAATCCTCCa	Cloning stuORF reporter with 20aa F
OLH52	ccggtGGAGGATTAGGAGGAGGAGGAGGAGGAGGAGGAGGAGGA GGAGGAGGAGGAGGAGGAGGAGGACATtttgtccggaCACa	Cloning stuORF reporter with 20aa R
OLH53	ctagtGTGtccggacaaaATGTCCTCCTCCTCCTCCTCCTCCTCCTCCTCCT CCTCCTCCTCCTCCTCCTCCTC CTCCTCCTCCTCCTCCTCCTCCTCCTCCTAATCCTCCa	Cloning stuORF reporter with 30aa F
OLH54	ccggtGGAGGATTAGGAGGAGGAGGAGGAGGAGGAGGAGGAGGA GGAGGAGGAGGAGGAGGAGGAGGAGGAGGAGGAGGAGGAGGA GGAGGAGGACATtttgtccggaCACa	Cloning stuORF reporter with 30aa R
OJB0498	CTAGTaagagaggcccaagATGgagacggcggcggctgtaGCCGCCtgacaggagccccA	Cloning aRaf uORF1 GCG to GCC mutations F
OJB0499	CCGGTggggctcctgtcaGGCGGCtacagccgccgccgtctcCATcttgggcctctcttA	Cloning aRaf uORF1 GCG to GCC mutations R
OJB0500	CTAGTgtccacggccaccATGgccTATTAGgggcagA	Cloning ATF4 uORF2 GCG to GCC mutation F
OJB0501	CCGGTctgcccCTAATAggcCATggtggccgtggacA	Cloning ATF4 uORF2 GCG to GCC mutation F
OJB0102	gatcGCTAGC ATGaccgaaatgagcttcct	ATF4 CDS into pCDNA3.1 F
OJB0103	gatcAAGCTT CTAggggacccttttcttcc	ATF4 CDS into pCDNA3.1 R

Supplementary Table 2: siRNA sequences

Target	Sequence or Product
DENR siRNA Pool	Dharmacon siGENOME SO-2705987G
eIF2D siRNA Pool	Dharmacon ON-TARGETplus SO-2780112G
c-Raf siRNA Pool	Dhamacon siGENOME SO-2766192G
ATF4 siRNA Pool	Dharmacon siGENOME SO-2757722G
a-Raf siRNA Pool	Dharmacon siGENOME SO-2759562G
MCTS1 siRNA Pool	Dharmacon siGENOME D-020590-04-0002

Supplementary Table 3: Antibodies used in this study

Antigen	Provider and Product Number	Blotting Dilution
c-Raf/Raf1	Cell Signaling #9422	1:1000
a-Raf	Cell Signaling #75804	1:1000
ATF4	Cell Signaling #11815	1:500
ASNS	Thermo Fisher PA5-56113	1:1000
Tubulin	T9026 Sigma	1:2500
RPS15	Atlas HPA054510	1:250
p-eIF2S1	Cell Signaling 3398S	1:1000
p-eIF2S1	Abcam ab32157S	1:1000
eIF2S1	Cell Signaling #5324	1:1000
MEK	Cell Signaling #9126	1:1000
p-MEK	Cell Signaling #9154	1:1000
ERK	Cell Signaling 4695	1:1000

p-ERK	Cell Signaling 4370	1:1000
DENR	Teleman Lab	1:1000
MCTS1	Teleman Lab	1:1000
eIF2D	Abcam ab108218	1:1000
HSP90	Cell Signaling #4877	1:1000
CDK4	Cell Signaling #12790	1:1000
p-S6K	Cell Signaling 9205L	1:1000
S6K	Cell Signaling 9202	1:1000

Supplementary Table 4: q-RT-PCR Primers

**Target**	**Sequence**
ATF4 forward	CCAACAACAGCAAGGAGGAT
ATF4 reverse	AGAAGGTCATCTGGCATGGTTTC
ASNS forward	TGAGGAAGGCATTCAGGCTC
ASNS reverse	CAGAGAAGATCACCACGCTAT
Actin B forward	CCACCATGTACCCTGGCATT
Actin B reverse	CGCTCAGGAGGAGCAATGAT

(continued on next page)

2) SUPPLEMENTARY FIGURES

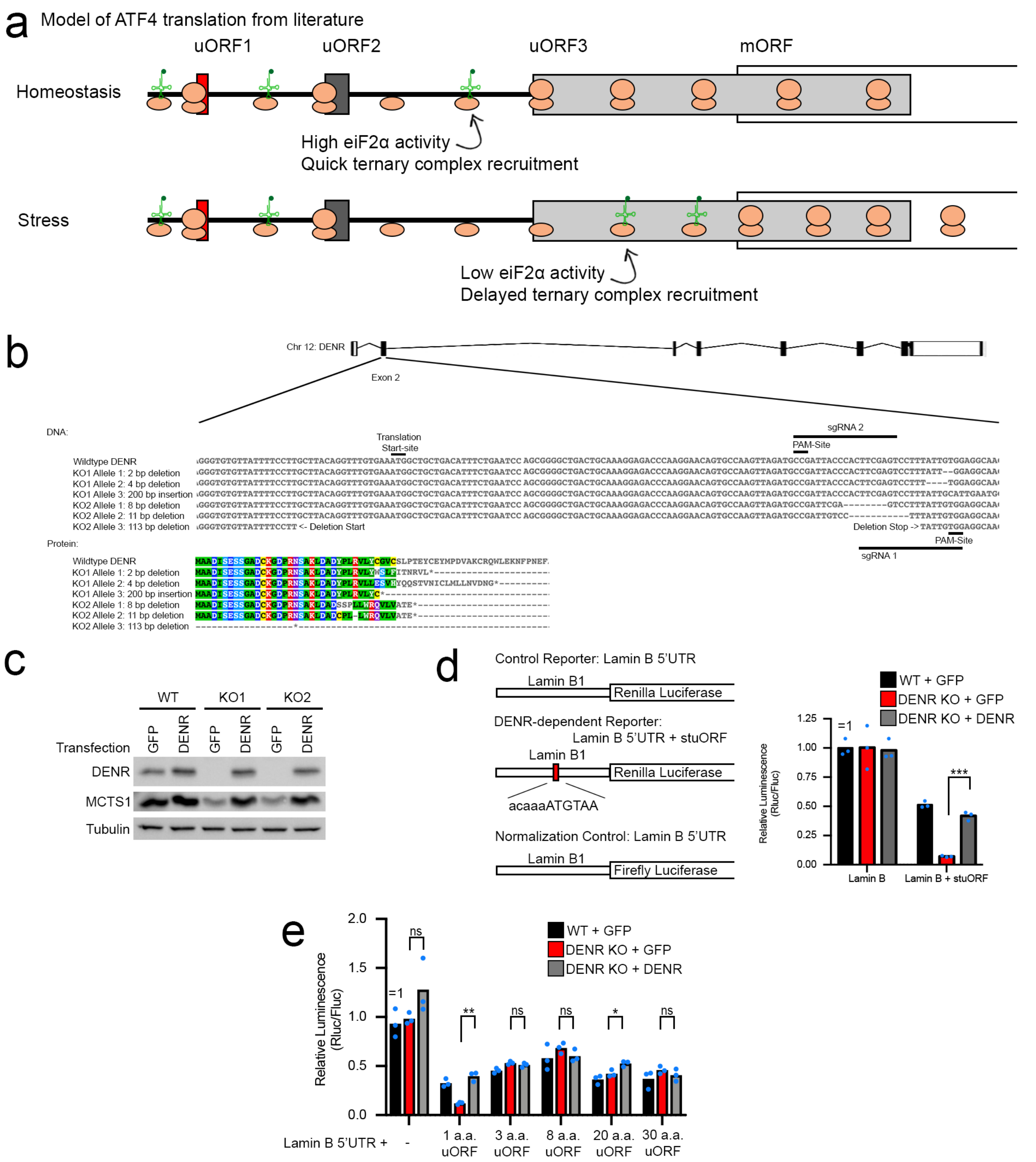

Supplementary Figure 1: Generation and phenotypic characterization of DENR^{KO} cells.

(a) Model of ATF4 translation. In non-stressed conditions, p-eIF2α levels are low and reinitiating 40S ribosomes from uORFs 1 and 2 are quickly reloaded with ternary-complexes.

Therefore, translation reinitiates at uORF3 and ATF4 translation is repressed. During stress, when p-eIF2α levels are higher, ternary complex activity is low, and reinitiating 40S ribosomes scan past uORF3 and initiate translation on the ATF4 main ORF.

(b) Molecular genotyping of DENR^{KO} cells. Mutated DNA and protein sequences are shown. Both sgRNAs target exon 2 shortly after the translation start site. HeLa cells have three copies of chromosome 12[68].

(c) Validation of DENR^{KO} HeLa cells. Western blot of two independent DENR^{KO} and control HeLa cell lines transfected with either GFP or DENR expression plasmid. Results are representative of three biological replicates.

(d) Dual-Luciferase reporter assay in HeLa control and DENR^{KO} cell lines for reinitiation after a short upstream ORF with a strong kozak sequence (stuORF). Cells were transfected with either a negative control renilla luciferase reporter containing the 5'UTR of *Lamin B1*, which contains no uORFs, or a DENR-dependent 'stuORF' reporter in which a synthetic uORF with a strong Kozak sequence (acaaaATGTAA) was cloned into the *Lamin B1* 5'UTR. In all cases, a firefly luciferase normalization control carrying the same *Lamin B1* 5'UTR was co-transfected. Additionally, cells were transfected with either a DENR expression plasmid or a GFP expression plasmid as a negative control. Luciferase activity was assayed 20 hours after transfection. Results are representative of three biological replicates. Unpaired, two-sided, non-parametric t-test: ***p<0.0005. p-value: 0.000087.

(e) Translation reporters analogous to the reporters in (d), but containing poly(TCC^{Ser}) uORFs of increasing length as described in [37], tested for DENR-dependence. Results are representative of three biological replicates. Unpaired, two-sided, non-parametric t-test: *p<0.05, **p<0.005. Three technical replicates are shown. p-values from left to right: 0.15, 0.00066, 0.27, 0.16, 0.017, 0.29.

Source data, including molecular weight marker positions, are provided in the Source Data file.

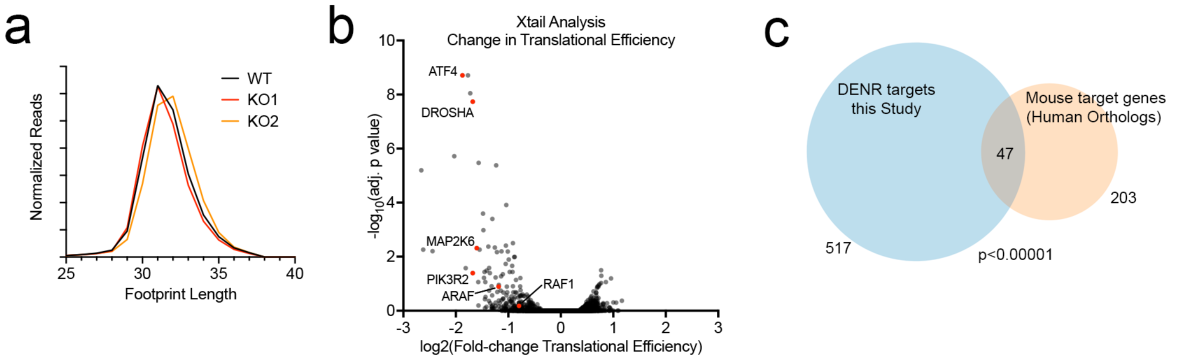

d

Downregulated Transcripts

PANTHER GO-Slim Molecular Function	n	Enrichment	Adj. p
RNA polymerase II proximal promoter sequence-specific DNA binding	9	4.23	0.0453
transcription regulatory region DNA binding	27	3.25	0.0003
transcription regulatory region sequence-specific DNA binding	18	2.94	0.0110
DNA binding	52	1.81	0.0085
protein kinase activity	36	2.12	0.0104
phosphotransferase activity, alcohol group as acceptor	42	2.07	0.0077
transferase activity, transferring phosphorus-containing groups	50	1.85	0.0102

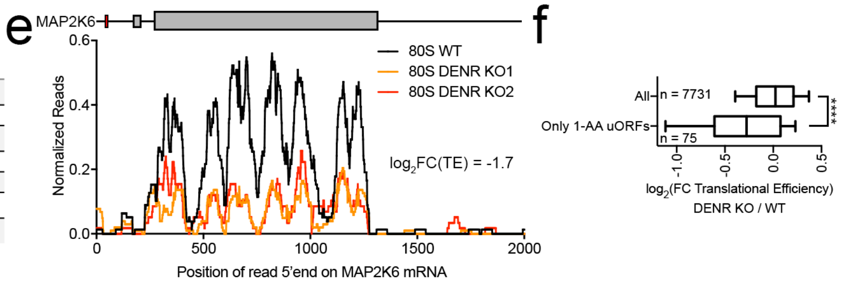

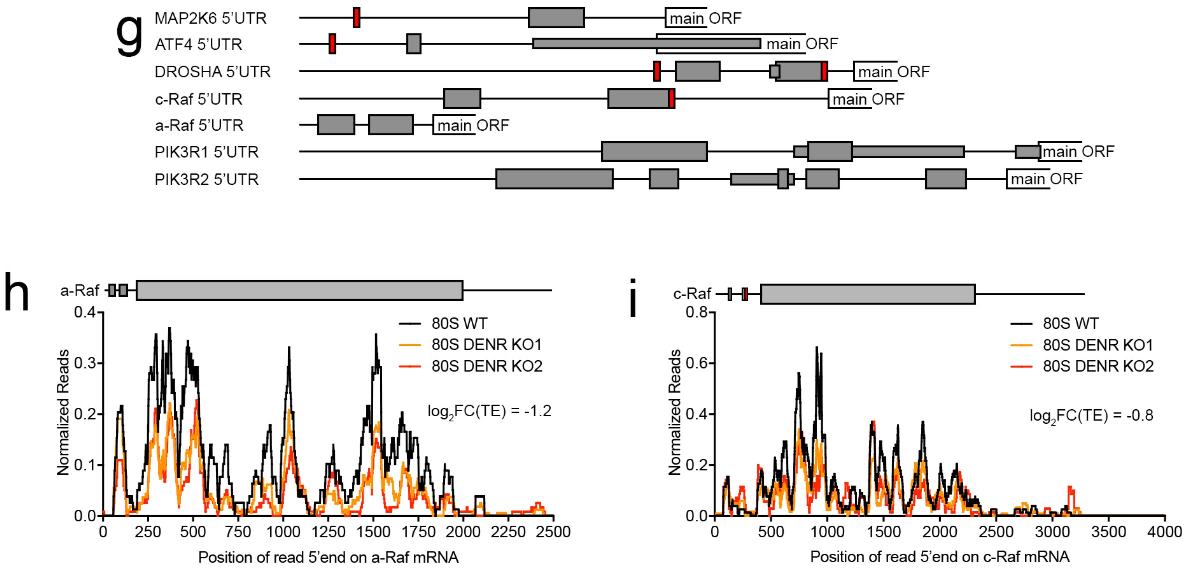

Supplementary Figure 2: Ribosome footprinting of DENR-KO cells.

(a) Average ribosome footprint length of control and $DENR^{KO}$ HeLa cells.

(b) Xtail analysis of changes in translation efficiency from 80S footprinting data of control and $DENR^{KO}$ HeLa cells identifies many transcripts with reduced translation efficiency and few with increased translation efficiency in $DENR^{KO}$ cells. Analogous to the z-vs-z analysis shown in main Figure 1c.

(c) Overlap between mouse DENR-targets from [44] and the DENR-targets identified here in HeLa cells. Mouse gene IDs were converted to their human orthologs using the Homologene NCBI database. Statistical significance of the overlap was assessed using binomial testing.

(d) Enrichment for molecular function Gene Ontology in the set of mRNAs that require DENR for optimal translation (Fig. 1c), determined by Panther GO enrichment analysis.

(e) 80S Ribosome occupancy on the *MAP2K6* transcript. Read counts were normalized to sequencing depth and scaled to mRNA abundance (one value for each cell line). Graphs were smoothened with a 50 nt sliding window. The $\log_2$ of fold-change in translation efficiency is also shown.

(f) Change in translational efficiency of all transcripts containing only 1AA uORFs (and no other uORFs) compared to all detected transcripts. **** $p < 10^{-6}$ non-parametric Kruskal-Wallis test corrected for multiple comparisons. Box middle = median, hinges = quartiles, whiskers = deciles.

(g) Schematic diagram of 5'UTR translation reporters of DENR targets. 5'UTR features: red = 1 AA uORF, grey = longer uORFs, Features are drawn to scale.

(h-i) Ribosome occupancy on the (h) *a-Raf* and (i) *c-Raf* transcripts. Read counts were normalized to sequencing depth and scaled to mRNA abundance (one value for each cell line). Graphs were smoothened with a 50nt sliding window. The $\log_2$ of fold-change in translation efficiency is also shown. 5'UTR features: red = 1 AA uORF, grey = longer uORFs. Features are drawn to scale.

Source data are provided as a Source Data file.

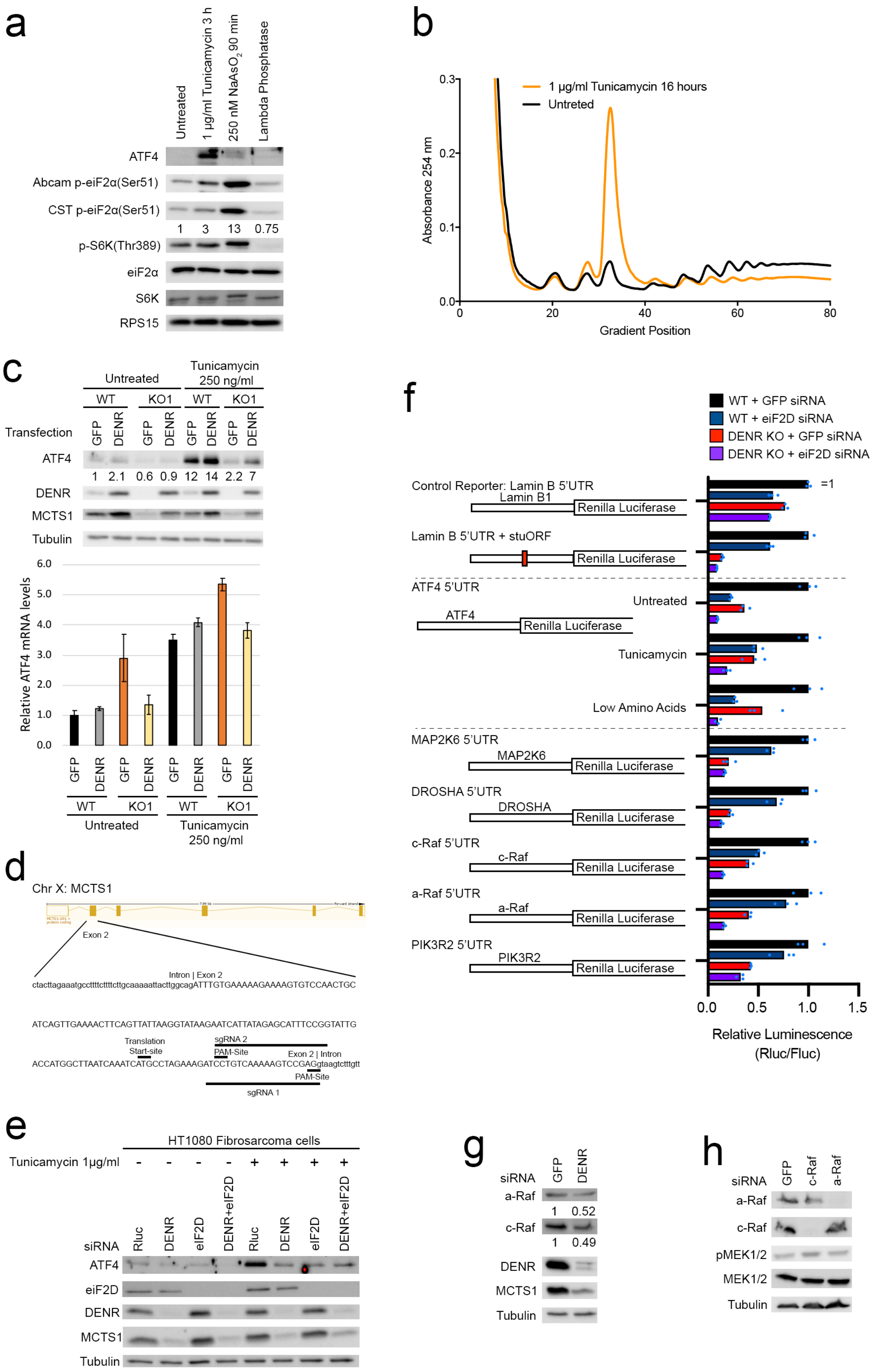

Supplementary Figure 3: Validation of new DENR targets
(legend continues on next page)

(a) p-eIF2S1(Ser51) antibodies detect unphosphorylated eIF2S1. HeLa cells treated with 1 μg/ml tunicamycin, 250 nM sodium arsenite or with vehicle for the indicated durations were harvested, and lysates were either boiled immediately in Laemmli buffer, or treated first with Lambda phosphatase for 30 minutes. p-eIF2α was quantified relative to total eIF2α. The anti-p-eIF2α antibody from Cell Signaling Technology was used in main Figures 2a-b.

(b) Tunicamycin treatment worked efficiently, judged by a global shutdown of protein translation. HeLa cells were treated with 1 μg/ml tunicamycin for 16 hours and then analysed on a 17.5%-50% sucrose gradient, where a strong disassembly of polysomes was observed.

(c) The defect in ATF4 induction in $DENR^{KO}$ cells is not due to a decrease in *ATF4* mRNA levels. Control or $DENR^{KO}$ HeLa cells were transfected with either GFP or DENR expression plasmid, re-seeded and treated with 1 μg/ml tunicamycin for 16 hours. q-RT-PCR for *ATF4* mRNA was carried out on total RNA (bottom panel) from the same cells as the western blot (bottom panel). mRNA levels were normalized to *Actin B* mRNA levels. Error bars show standard deviation of three technical replicates. Results are representative of three biological replicates.

(d) Scheme of sgRNAs targeting the *MCTS1* exon 2 on chromosome X.

(e) ATF4 protein levels are dependent on DENR and eIF2D in HT1080 cells. DENR and eIF2D were depleted using siRNAs. Cells were re-seeded after 72 hours of knockdown and treated with 1 μg/ml tunicamycin for 16 hours. Results are representative of three biological replicates.

(f) Knockdown of *eIF2D* has additive effects with DENR loss-of-function on translation reporters containing the 5'UTRs of genes identified as DENR targets by ribosomes footprinting. Same data as in Figure 2f but only normalized to the *Lamin B* control reporter in control cells. Results are representative of two biological replicates. Three technical replicates are shown.

(g) siRNA mediated *DENR* depletion decreases a-Raf and c-Raf protein levels in HeLa cells. Results are representative of three biological replicates.

(h) Verification of isoform specificity of a-Raf and c-Raf antibodies by siRNA mediated knockdown and immunoblotting.

Source data, including uncropped western blots with molecular weight marker positions, are provided in the Source Data file.

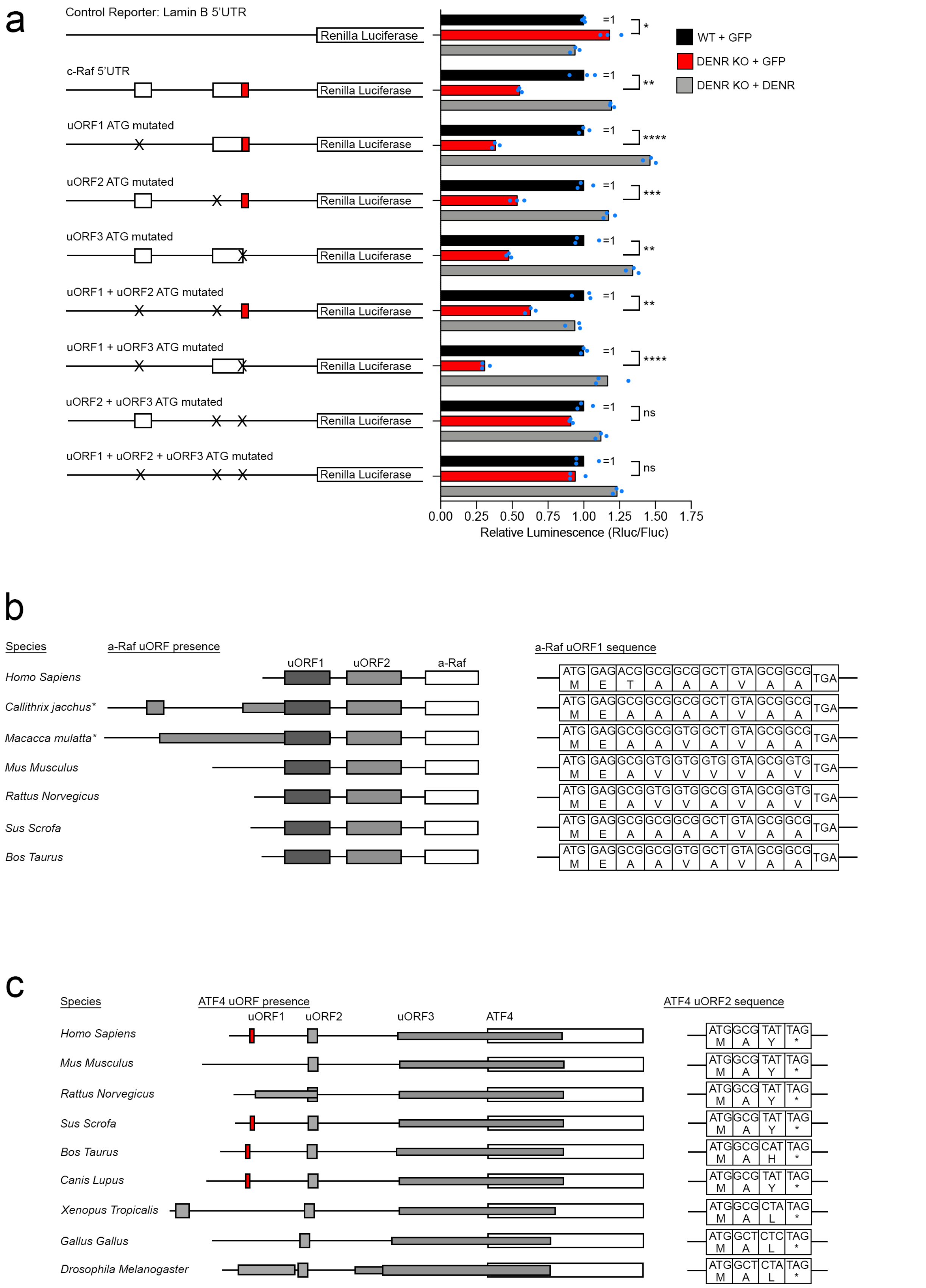

Supplementary Figure 4: Identification of DENR-dependent, long uORFs in the 5'UTRs of ATF4, a-Raf and c-Raf.

(legend continues on next page)

(a) uORF2 and nested uORF3 are DENR-dependent features in the *c-Raf* 5'UTR. Luciferase assay of reporters carrying the *c-Raf* 5'UTR with combinations of point mutations on the start codons (ATG to TAC) of the various uORFs, tested in control and $DENR^{KO}$ HeLa cells. Cells were additionally transfected with GFP or DENR expression plasmids as indicated. Unpaired, two-sided, non-parametric t-test: *p<0.05, **p<0.005 ***p<0.0005, ****p<0.00005. Results are representative of three biological replicates. Three technical replicates are shown. p-values from top to bottom: 0.014, 0.0011, 0.000021, 0.00047, 0.00066, 0.0014, 0.0000059, 0.053, 0.40.

(b) Distribution and sequence of *a-Raf* uORFs across animal species, from NCBI RefSeq mRNA sequences.

(c) Distribution and sequence of *ATF4* uORFs across animal species, from NCBI RefSeq mRNA sequences.

Source data are provided as a Source Data file.

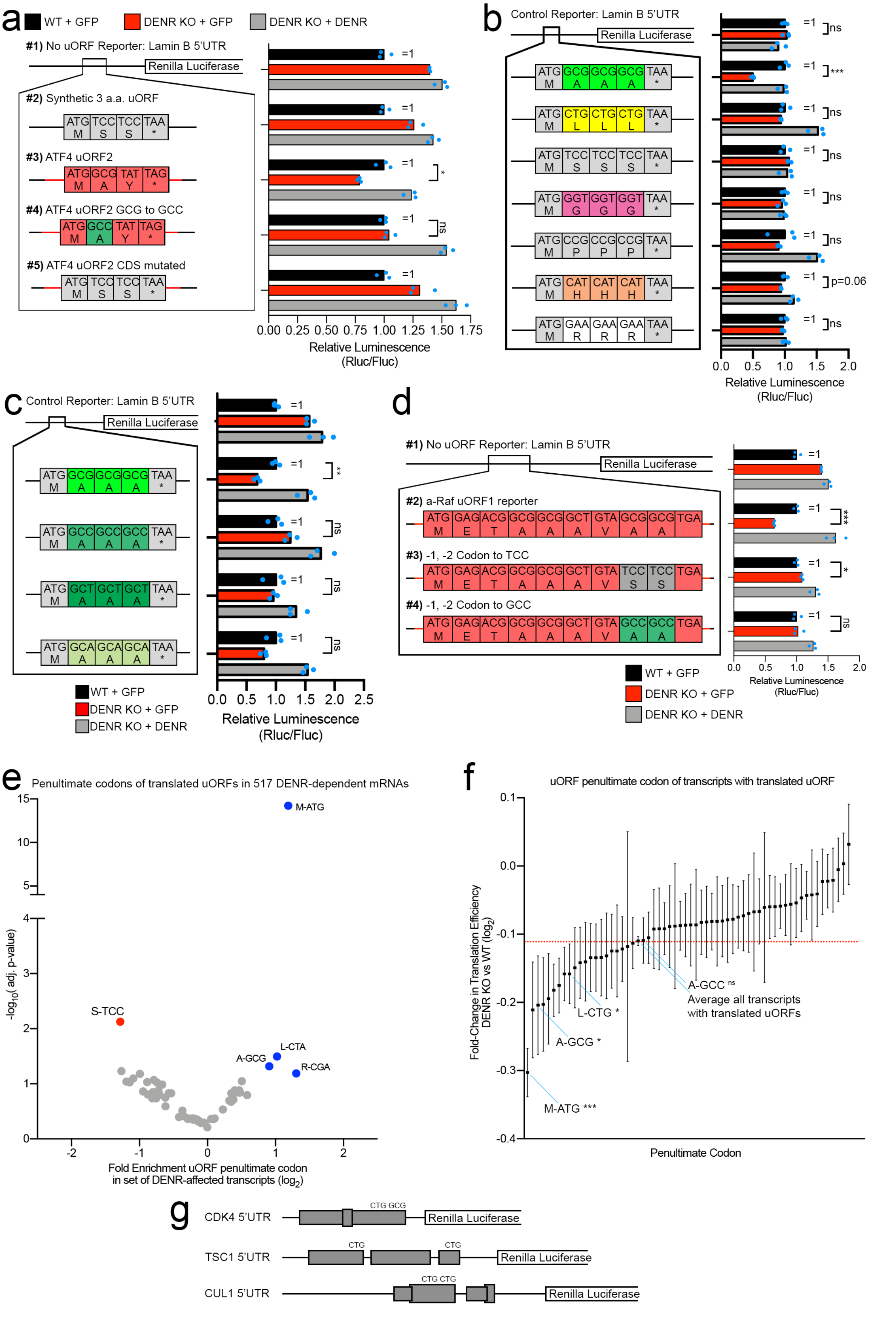

Supplementary Figure 5: Identification of codons dependent on DENR for reinitiation.
(legend continues on next page)

(a) Mutation of the codons of *ATF4* uORF2 removes DENR-dependence. Luciferase assay of reporters carrying mutated versions of *ATF4* uORF2, as indicated, cloned into the *Lamin B1* 5'UTR, tested in control and $DENR^{KO}$ HeLa cells. Cells were additionally transfected with GFP or DENR expression plasmids as indicated. Results are representative of three biological replicates. Unpaired, two-sided, non-parametric t-test: *p<0.05. Three technical replicates are shown. p-values from top to bottom: 0.0052, 0.27.

(b) DENR-dependence of various codons. Luciferase reporter assay of cells transfected with indicated reporter plasmids and either GFP or DENR overexpression plasmid. Normalized to an FLuc control reporter. Unpaired, two-sided, non-parametric t-test: ***p<0.0005. Results are representative of three biological replicates. Three technical replicates are shown. p-values from top to bottom: 0.51, 0.00031, 0.24, 0.23, 0.42, 0.53, 0.057, 0.42.

(c) Codon identity, but not the identity the amino acid encoded by the codon, determines DENR-dependence. A uORF with terminal GCG^{Ala} codons, but not GCC^{Ala} codons, is DENR dependent. Luciferase reporter assay of cells transfected with indicated reporter plasmids and either GFP or DENR overexpression plasmid. Normalized to an FLuc control reporter. Results are representative of three biological replicates. Unpaired, two-sided, non-parametric t-test: **p<0.005. Three technical replicates are shown. p-values from top to bottom: 0.0026, 0.053, 0.75, 0.078.

(d) Conversion from Ala^{GCG} to Ala^{GCC} of the last two codons of *a-Raf* uORF1 removes DENR-dependence. Luciferase assay of reporters carrying mutated versions of a-Raf uORF1, as indicated, cloned into the *Lamin B1* 5'UTR, tested in control and $DENR^{KO}$ HeLa cells. Cells were additionally transfected with GFP or DENR expression plasmids as indicated. Results are representative of three biological replicates. Unpaired, two-sided, non-parametric t-test: *p<0.05, ***p<0.0005. Three technical replicates are shown. p-values from top to bottom: 0.00011, 0.016, 0.74.

(e) mRNAs with reduced translation efficiency in DENR^{KO} cells have uORFs that are enriched for particular penultimate codons, such as M-ATG, L-CTG or A-GCG. Enrichment of penultimate codons in the translated uORFs of the 517 DENR-dependent mRNAs (main Fig. 1c) compared to all translated uORFs genome-wide. Statistical significance of the enrichment was calculated using a binomial distribution, adjusted for multiple testing (61 tests, one for each amino acid coding nucleotide triplet).

(f) Genome-wide, transcripts that contain uORFs with particular penultimate codons show reduced translation efficiency in DENR^{KO} cells. The average log2(fold-change translation efficiency) in DENR^{KO} cells for all transcripts containing translated uORFs with the indicated penultimate codons is shown. Transcripts containing multiple uORFs were counted multiple times. Dot = mean, error bars = 95% confidence intervals. Statistical significance was assessed with Mann-Whitney test, *p-value < 0.05, ***p-value < 0.0005.

(g) Schematic diagram of 5'UTR translation reporters of predicted DENR targets. 5'UTR features: grey = uORFs, Features are drawn to scale.

Source data are provided as a Source Data file.

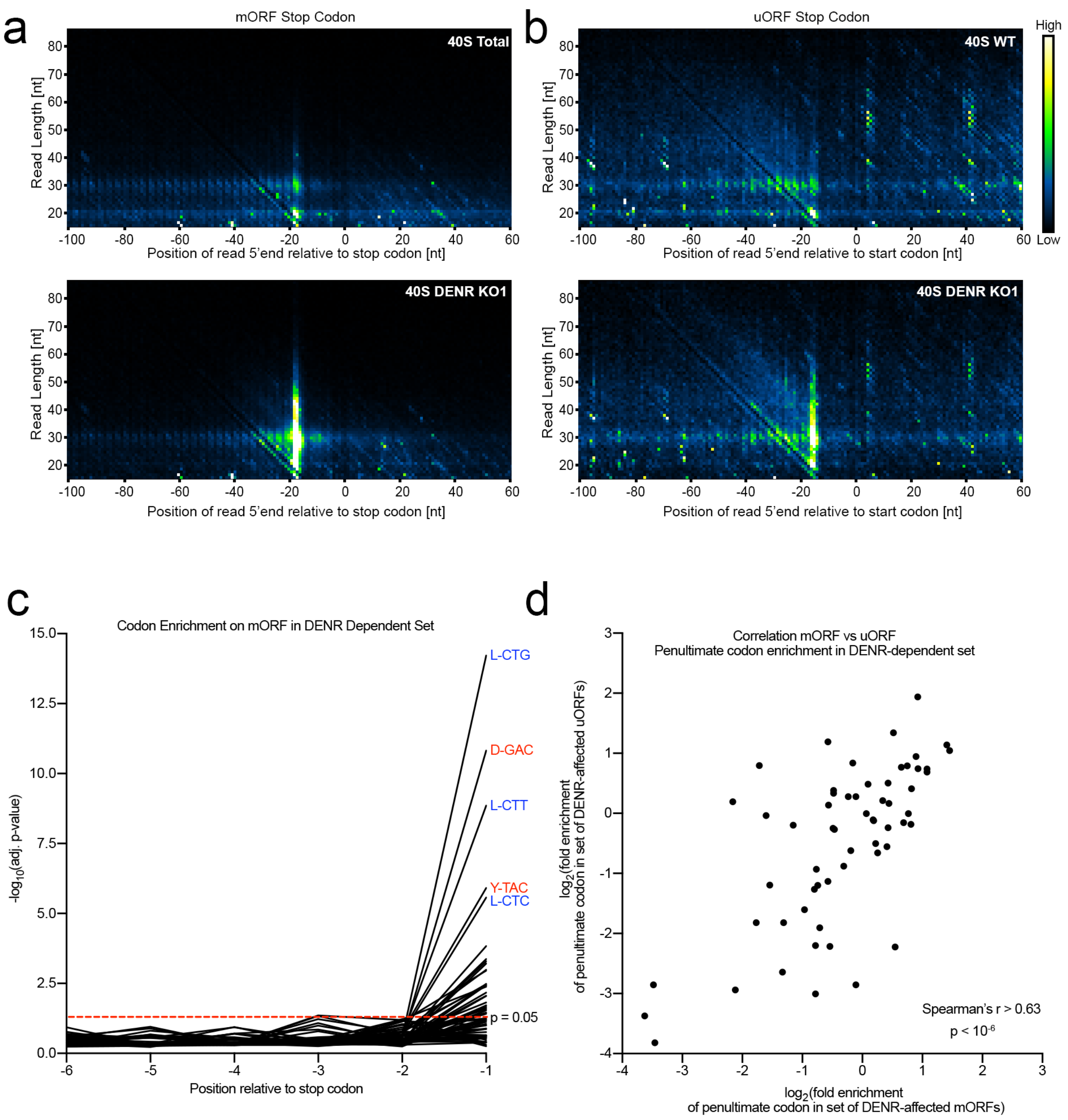

Supplementary Figure 6: 40S Ribosome footprinting reveals codon-specific ribosome recycling defects in DENR^{KO} cells.

(a-b) Post-termination 40S ribosomes can be seen on stop-codons genome wide, and these recycling ribosomes stall in DENR^{KO} cells, as can be seen by an increased abundance of these 40S intermediates. 2-dimensional metagene plots, resolving footprint position on the x-axis and footprint length on the y-axis, of 40S ribosome footprints aligned to main ORF (a) or uORF

(b) stop codons in control (top) and DENR^{KO} (bottom) cells. The number of reads is displayed according to a linear color scale, shown on the right and normalized to sequencing depth.

(c) Post-termination 40S ribosomes stall on stop codons in DENR^{KO} cells when the ORF contains particular penultimate codons (position -1) but not other terminal codons (positions -2 to -6). All main ORFs were classified as either DENR-independent (n = 6566) or DENR-dependent (n=1222) for 40S recycling, depending on whether they have increased 40S footprint counts on their stop codons in DENR^{KO} cells compared to control HeLa cells. Codon enrichment was then calculated in the DENR-dependent set compared to the DENR-independent set, and statistical significance of enrichment was assessed using binomial tests, adjusted for multiple testing.

(d) The same penultimate codons that cause post-termination 40S ribosomes to stall on the stop codons of main ORFs in DENR^{KO} cells also do so when they are present in uORFs. All main ORFs (mORF) and uORFs were classified as either DENR-independent or DENR-dependent for 40S recycling, depending on whether they have increased 40S footprint counts on their stop codons in DENR^{KO} cells compared to control HeLa cells. The enrichment for each of the possible 61 penultimate codons in the DENR dependent sets (mORFs or uORFs separately) were calculated compared to the DENR-independent sets. Each point represents one codon, with enrichment in mORFs on the x-axis and enrichment in uORFs on the y-axis. Goodness of correlation was assessed by Spearman's r; p-value$<10^{-6}$.

Source data are provided as a Source Data file.

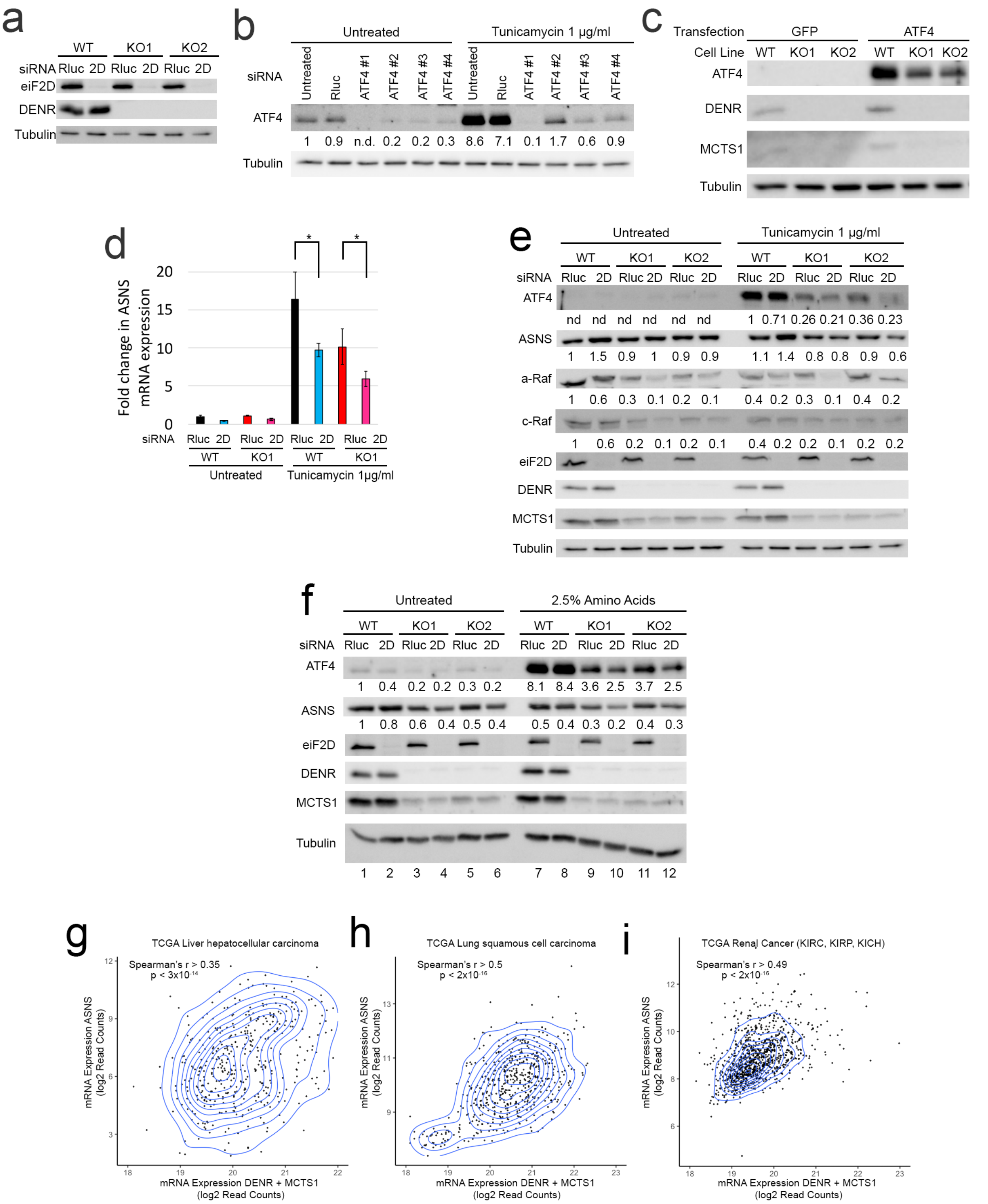

Supplementary Figure 7: ATF4 is a DENR-dependent target in HeLa cells and human cancers

(a) Knockdown efficiency of eIF2D in control and DENR^{KO} HeLa cells used for the proliferation assay in Fig. 5b.

(b) Knockdown efficiency of ATF4 in HeLa cells used for the proliferation assay in Fig. 5c.

(c) ATF4 overexpression efficiency in control and $DENR^{KO}$ HeLa cells used for the proliferation assay in Fig. 5d.

(d-e) DENR/MCTS1 and eIF2D are required for optimal ATF4 translation and target gene induction. (d) q-RT-PCR for mRNA levels of *ASNS*, a direct ATF4 target, normalized to *Actin B* mRNA (n=3 technical replicates). (e) Western blot analysis of control or $DENR^{KO}$ cells treated with 1 µg/ml tunicamycin for 16 hours. Cells were transfected with siRNA targeting either renilla luciferase (negative control) or eIF2D. Quantification of ATF4 and ASNS band intensity is normalized to tubulin. Cells are from the same experiment as in (d). Data are presented as mean +/- SD of 3 technical replicates. Representative of 2 biological replicates.

(f) ATF4 and ASNS expression are DENR and eIF2D dependent during amino acid starvation. Western blot analysis of control or $DENR^{KO}$ cells treated with low amino acids (2.5% of DMEM) for 16 hours. Cells were transfected with siRNA targeting either renilla luciferase (negative control) or eIF2D. Quantification of ATF4 and ASNS band intensity is normalized to RPS15. Results are representative of three biological replicates.

(g) *DENR+MCTS1* and *ASNS* mRNA levels correlate across liver hepatocellular carcinoma samples. Correlation, using Xena [67], of TCGA mRNA expression data. Shown is spearman's r and p-value. n=423. p-value = $3*10^{-14}$.

(h) *DENR+MCTS1* and *ASNS* mRNA levels correlate across lung squamous cell carcinoma samples. Correlation, using Xena [67], of TCGA mRNA expression data. Shown is spearman's r and p-value. n=553. p-value < $2.2x10^{-16}$.

(i) *DENR+MCTS1* and *ASNS* mRNA levels correlate across renal cancer samples. Correlation, using Xena [67], of TCGA mRNA expression data. Shown is spearman's r and p-value. n=1020. p-value < $2.2x10^{-16}$.

Source data, including molecular weight marker positions, are provided in the Source Data file.

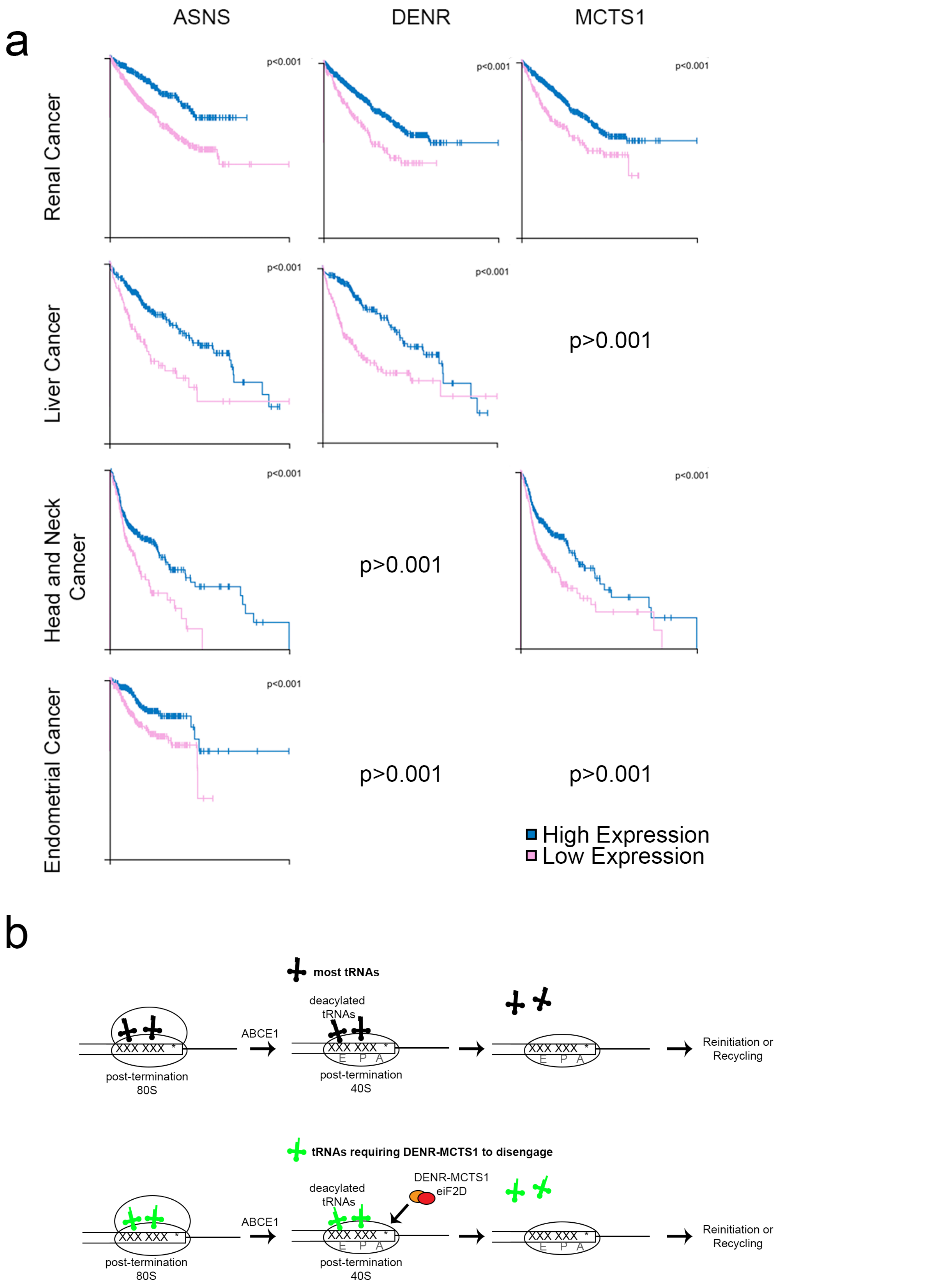

Supplementary Figure 8: DENR and ASNS expression correlate in normal tissue and cancer, and a model for how DENR•MCTS1 promote 40S ribosome recycling.
(legend continues on next page)

(a) Prognostic value of *DENR*, *MCTS1* and *ASNS* mRNA expression in certain cancers. Analysis by ProteinAtlas.org of TCGA data. The cutoff for assigning samples to the high expression or low expression groups is the median of the dataset. p-values: Renal Cancer (left to right): $6.2*10^{-7}$, 0.0000086, 0.000043 Liver Cancer (left to right): 0.0000094, $1.6*10^{-7}$ Head and Neck Cancer (left to right): 0.000088, 0.000033 Endometrial Cancer: 0.00042

(b) Model of how DENR•MCTS1 and eIF2D promote post-termination 40S ribosome recycling. After translation termination, peptide release and subunit splitting, the post termination 40S ribosome contains 1 or 2 deacylated tRNAs (in the E- and P-site). Only certain tRNAs require DENR•MCTS1 or eIF2D for disengagement. In these cases, DENR•MCTS1 and/or eIF2D are needed for subsequent reinitiation or recycling.
