# Supplementary material for: DENR promotes translation reinitiation via ribosome recycling to drive expression of oncogenes including ATF4
Source: Nat Commun. 2020 Sep 16;11:4676. doi: 10.1038/s41467-020-18452-2 (PMC7494916; doi:10.1038/s41467-020-18452-2)
Supplement: Supplementary file 3 — Reporting Summary [file 41467_2020_18452_MOESM3_ESM.pdf]

nature research

Corresponding author(s): Aurelio Teleman
Last updated by author(s): Jul 22, 2020

Reporting Summary

Nature Research wishes to improve the reproducibility of the work that we publish. This form provides structure for consistency and transparency in reporting. For further information on Nature Research policies, see Authors & Referees and the Editorial Policy Checklist.

Statistics

For all statistical analyses, confirm that the following items are present in the figure legend, table legend, main text, or Methods section.

n/a	Confirmed	
☐	☒	The exact sample size (*n*) for each experimental group/condition, given as a discrete number and unit of measurement
☐	☒	A statement on whether measurements were taken from distinct samples or whether the same sample was measured repeatedly
☐	☒	The statistical test(s) used AND whether they are one- or two-sided<br>*Only common tests should be described solely by name; describe more complex techniques in the Methods section.*
☒	☐	A description of all covariates tested
☐	☒	A description of any assumptions or corrections, such as tests of normality and adjustment for multiple comparisons
☐	☒	A full description of the statistical parameters including central tendency (e.g. means) or other basic estimates (e.g. regression coefficient) AND variation (e.g. standard deviation) or associated estimates of uncertainty (e.g. confidence intervals)
☒	☐	For null hypothesis testing, the test statistic (e.g. *F*, *t*, *r*) with confidence intervals, effect sizes, degrees of freedom and *P* value noted<br>*Give P values as exact values whenever suitable.*
☒	☐	For Bayesian analysis, information on the choice of priors and Markov chain Monte Carlo settings
☒	☐	For hierarchical and complex designs, identification of the appropriate level for tests and full reporting of outcomes
☒	☐	Estimates of effect sizes (e.g. Cohen's *d*, Pearson's *r*), indicating how they were calculated

Our web collection on statistics for biologists contains articles on many of the points above.

Software and code

Policy information about availability of computer code

Data collection	Software that runs the BioRad Chemidoc is commercially available.
Data analysis	Bowtie 2 version 2.3.4.2<br>Cutadapt 2.3 with Python 3.6.4<br>BBMap version 37.93<br>Microsoft Excel<br>Graphpad Prism 7<br>Custom Scripts for plotting mapped deep-sequencing reads are available at github: https://github.com/aurelioteleman/Teleman-Lab

For manuscripts utilizing custom algorithms or software that are central to the research but not yet described in published literature, software must be made available to editors/reviewers. We strongly encourage code deposition in a community repository (e.g. GitHub). See the Nature Research guidelines for submitting code & software for further information.

Data

Policy information about availability of data

All manuscripts must include a data availability statement. This statement should provide the following information, where applicable:

- Accession codes, unique identifiers, or web links for publicly available datasets
- A list of figures that have associated raw data
- A description of any restrictions on data availability

All high-throughput sequencing data presented in this study is available at: NCBI Geo: GSE140084

Field-specific reporting

Please select the one below that is the best fit for your research. If you are not sure, read the appropriate sections before making your selection.

☒ Life sciences ☐ Behavioural & social sciences ☐ Ecological, evolutionary & environmental sciences

For a reference copy of the document with all sections, see nature.com/documents/nr-reporting-summary-flat.pdf

Life sciences study design

All studies must disclose on these points even when the disclosure is negative.

Sample size	No statistical methods were used to determine sample size, but based on experience three biological replicates were used for experiments with standard methods.
Data exclusions	No Data were excluded
Replication	Only reproducible data are included in the manuscript
Randomization	Not applicable
Blinding	No blinding was done

Reporting for specific materials, systems and methods

We require information from authors about some types of materials, experimental systems and methods used in many studies. Here, indicate whether each material, system or method listed is relevant to your study. If you are not sure if a list item applies to your research, read the appropriate section before selecting a response.

Materials & experimental systems

n/a	Involved in the study	
☐	☒	Antibodies
☐	☒	Eukaryotic cell lines
☒	☐	Palaeontology
☒	☐	Animals and other organisms
☒	☐	Human research participants
☒	☐	Clinical data

Methods

n/a	Involved in the study	
☒	☐	ChIP-seq
☒	☐	Flow cytometry
☒	☐	MRI-based neuroimaging

Antibodies

Antibodies used	All antibodies used are listed in supplemental table 1 of the manuscript.
Validation	DENR and MCTS1 antibodies are validated in figure 2a.<br>c-Raf and a-Raf antibodies are validated in supplemental figure 2e<br>All other antibodies are standard, commercial antibodies that are widely used in the field.

Eukaryotic cell lines

Policy information about cell lines

Cell line source(s)	HT1080 Cells were provided by Stefan Pusch (DKFZ), Hela cells were provided by Micheal Boutros Lab (DKFZ). Jurkat cells were purchased from DSMZ
Authentication	Hela and HT1080 cells were authenticated by multiplexion inc. (Heidelberg), Jurkat cells were authenticated by DSMZ.
Mycoplasma contamination	Hela and HT1080 cells tested negative for mycoplasma contamination, Jurkat cells were not tested in lab, but were tested by DSMZ.
Commonly misidentified lines<br>(See ICLAC register)	*Name any commonly misidentified cell lines used in the study and provide a rationale for their use.*
