# Supplementary material for: DENR promotes translation reinitiation via ribosome recycling to drive expression of oncogenes including ATF4
Source: Nat Commun. 2020 Sep 16;11:4676. doi: 10.1038/s41467-020-18452-2 (PMC7494916; doi:10.1038/s41467-020-18452-2)
Supplement: Supplementary file 4 — Description of Additional Supplementary Files [file 41467_2020_18452_MOESM4_ESM.pdf]

**Description of Additional Supplementary Files**

File Name: Supplementary Data 1
Description: Translation Efficiency for 15722 transcripts in DENR WT and KO cells.

File Name: Supplementary Data 2
Description: Change in 40S stop codon occupancy on main ORFs in DENR KO cells.

File Name: Supplementary Data 3
Description: Change in 40S stop codon occupancy on uORFs in DENR KO cells.
